# Long-Term Neurological Impact of COVID-19

**DOI:** 10.7759/cureus.18131

**Published:** 2021-09-20

**Authors:** Jitesh Kumar, Kainat Makheja, FNU Rahul, Suneel Kumar, Manoj Kumar, Momal Chand, Yasir Kammawal, Dua Khalid, Maha Jahangir, Parkash Bachani

**Affiliations:** 1 Internal Medicine, Ghulam Muhammad Mahar Medical College, Sukkur, PAK; 2 Internal Medicine, Jinnah Sindh Medical University, Karachi, PAK; 3 Neurology, South City Hospital, Karachi, PAK; 4 Internal Medicine, Dow University of Health Sciences, Karachi, PAK; 5 Pathology, Ascension St. John Hospital, Detroit, USA; 6 Internal Medicine, Baqai Medical University, Karachi, PAK; 7 Internal Medicine, Liaquat University of Medical and Health Sciences, Jamshoro, PAK

**Keywords:** sars-cov-2 (severe acute respiratory syndrome coronavirus -2), covid-19, neurological outcomes, cns disorders, loss of smell

## Abstract

Introduction: Recent research has observed the ability of coronavirus disease 2019 (COVID-19) to spread in the brain from the respiratory system. The associated neurological disorder includes encephalopathies, inflammatory syndromes, stroke, peripheral neuropathies, and various other central nervous system disorders. This study aims to highlight the long-term neurological sequelae in patients with COVID-19 disease.

Methods: This long-term study was carried out in the COVID-19 unit of a tertiary care hospital in Pakistan from July 2020 to July 2021. After obtaining informed consent, we enrolled 1000 patients who recovered from COVID-19 and were discharged. The participants were followed up after 30 and 90 days.

Results: At the time of enrollment, there were 602 (60.2%) males and 398 (39.8%) females. The most common neurological symptom on 30-day follow-up was headache (8.8%), followed by insomnia. The most common neurological symptom on day 90 follow-up was insomnia (5.07%), followed by an altered sense of smell (3.3%).

Conclusion: COVID-19 tends to produce a wide range of neurological symptoms, ranging from headache to anosmia to increased risk of stroke, that complicates clinical management. Potential neurologic effects and drug interactions have been reported secondary to the medications used to treat COVID-19. In light of the aforementioned facts, COVID-19 could potentially have a long-term effect on the brain. Therefore, it is important that the clinicians must be aware of the potential neurologic complications. Lastly, proper follow-up is recommended that would aid in timely recognition and management of the neurological disorder.

## Introduction

Coronavirus disease 2019 (COVID-19) is a global pandemic declared by the World Health Organization on March 11, 2020 [1]. It is caused by severe acute respiratory syndrome coronavirus 2 (SARS-CoV-2). In December 2019, the outbreak of this novel disease started in Wuhan city of China which then spread to other parts of the world [2]. It particularly involves the lower respiratory tract system [3]. The most common symptoms of COVID-19 are fever, fatigue, dry cough, myalgia, and dyspnea, while less common ones are headache, abdominal pain, diarrhea, nausea, and vomiting [4]. Some severe and complicated cases can cause respiratory failure and multiorgan failure with high mortality [5]. Recent research also observed that SARS-CoV-2 has the ability to spread in the brain from the respiratory system [6]. It is also a possibility that the virus acts as an initiating agent of some neurodegenerative diseases like Parkinson’s disease in the long term [7]. According to recent research, 36% of the COVID-19 patients suffered from neurological symptoms, while 25% of them can be due to direct brain and spinal cord invasion. The major clinical features include dizziness, headache, altered consciousness, and seizures [8]. Critical patients also showed agitation, confusion, and corticospinal tract signs like enhanced tendon reflex and clonus [9].

COVID-19-associated neurological disorder has been categorized into five types which include encephalopathies, inflammatory syndromes, stroke, peripheral neuropathies, and various other central nervous system (CNS) disorders [10]. Research conducted in the UK emphasized that persistent neurological symptoms remained after COVID-19 infection [11]. However, the pathophysiology is associated with direct invasion of the virus into the brain or virus-induced hyperinflammatory and hypercoagulable states and postinfectious immune-mediated processes. However, hypoxemia and endothelium dysfunction due to COVID-19 can also cause neurological damage in the long term, thus it is essential to monitor the patients to understand the potential neurological complications [12].

Although it has been observed that some patients totally recover from COVID-19 pneumonia, the disease's long-term effects and complications on the CNS still need to be explored. Thus, the main aim is to provide enough evidence and data on the mechanisms involved in the development of the long-term neurological sequelae of SARS-CoV-2 infection. Furthermore, the risk factors that lead to the development of these neurological manifestations should be explored. Thus, it is the need of the hour to further investigate to determine neurological manifestations caused by COVID-19 disease. Therefore, this study aims to highlight the long-term neurological sequelae in patients with COVID-19 disease.

## Materials and methods

This long-term study was carried out in the COVID-19 unit of a tertiary care hospital in Pakistan from July 2020 to July 2021. We enrolled 1000 patients who recovered from COVID-19 and were discharged. Informed consent was obtained before their enrollment in the study. The sampling technique used was consecutive convenient non-probability sampling. Before the enrollment of participants, ethical review board approval was taken from Ghulam Muhammad Mahar Medical College (GMMMC/IRB/2020-COVID-19/03). The participants were informed to get a check-up after 30 and 90 days.

Upon discharge, their neurological symptoms were noted in a self-structured questionnaire. At follow-up, participants were inquired about neurological symptoms such as altered sense of smell, taste, and vision, headache, insomnia, seizures, dizziness, and cerebrovascular events. Participants who could not come for follow-up were inquired about their symptoms via phone call. A total of 183 participants were lost to follow-up, 71 on day 30 and 112 participants on day 90.

Data were analyzed using the Statistical Package for the Social Sciences, v.22.0 (SPSS; IBM Corp., Armonk, NY, United States). For categorical data, frequency and percentage were calculated. Mean and standard deviation were calculated for numerical data. A p-value of less than 0.05 meant that there is a significant difference in the value between the two groups and the null hypothesis is void.

## Results

The mean age of participants was 41 ± 9 years. At the time of enrollment, there were 602 (60.2%) males and 398 (39.8%) females (Table 1).

**Table 1:** Characteristics of participants at the time of discharge CRP: C-reactive protein, ESR: erythrocyte sedimentation rate, IU: international unit, LDH: lactate dehydrogenase, mg/L: milligrams per litre, mm/hr: millimeters per hour

Characteristics at the time of discharge	Mean ± SD (n=1000)
Age (in years)	41 ± 9
Gender	
Male	602 (60.2%)
Female	398 (39.8%)
Mean number of days at the hospital	4.2 ± 1.2
CRP (mg/L)	12.2 ± 3.5
LDH (IU)	307.6 ± 82.5
ESR (mm/hr)	13.1 ± 4.1

On day 0 (at discharge), the most common neurological symptom was headache (11.2%), followed by altered sense of smell (7.1%) (Table 2).

**Table 2:** Neurological manifestations on day 0-Discharge

Symptoms	Frequency
Headache	112 (11.2%)
Insomnia	42 (4.2%)
Altered Sense of Smell	71 (7.1%)
Altered Sense of Taste	52 (5.2%)
Altered Vision	48 (4.8%)
Dizziness	50 (5.0%)

On day 30, 929 participants returned for follow-up, which included 571 (61.5%) males and 358 (38.5%) females. The most common neurological symptom on 30-day follow-up was headache (8.8%), followed by insomnia (5.5%) (Table 3).

**Table 3:** Neurological manifestations on day 30 follow-up

Symptoms	Frequency
Headache	82 (8.8%)
Insomnia	52 (5.5%)
Altered sense of smell	36 (3.8%)
Altered sense of taste	32 (3.4%)
Altered vision	26 (2.7%)
Dizziness	25 (2.6%)
Stroke	01 (0.1%)

On day 90, 817 participants returned for follow-up, 512 (62.66%) were males and 305 (37.33%) were females. The most common neurological symptom on day 90 follow-up was insomnia (5.07%), followed by an altered sense of smell (3.3%) (Table 4).

**Table 4:** Neurological manifestations on day 90 follow-up

Symptoms	Frequency
Insomnia	41 (5.07%)
Altered sense of smell	27 (3.3%)
Headache	22 (2.6%)
Altered sense of taste	18 (2.2%)
Altered vision	16 (1.9%)
Dizziness	15 (1.8%)
Stroke	03 (0.3%)

## Discussion

Our study showed that on 30-day follow-up, the most common neurological symptom was headache (8.8%), followed by insomnia (5.5%). However, participants on 90-day follow-up reported insomnia (5.07%), followed by an altered sense of smell (3.3%) frequently. In concordance with our results, a study including 357 patients demonstrated that 85.6% had olfaction-related problems caused by COVID-19. Among them, 79.6% reported complete loss of smell while 20.4% reported a partial loss of smell [13]. This could be due to the olfactory neurons being attacked in the olfactory epithelium. Moreover, cells in the olfactory epithelium express angiotensin-converting enzyme 2 (ACE2) and transmembrane protease, serine 2 (TMPRSS2) protein receptors that are needed for the progression of COVID-19 in the body [14,15].

A study from Wuhan supporting our results suggested that among the 36.4% COVID-19 patients with neurological symptoms, headache (13.1%) was one of the most frequently observed symptoms [8]. Another prospective analysis from Wuhan showed that 8% of the patients experienced headache, which is considered to be the most frequently reported neurological manifestation [16]. Keeping the problem of insomnia in mind, several studies concluded that insomnia was faced significantly by the people affected by COVID-19 as compared to the rest of the population [17-19].

In order for SARS-CoV-2 to attack, it is thought to require both a cell surface receptor for the viral spike (S) protein to tie to, in addition to the preparation of the S protein by cell proteases. In particular, SARS-CoV-2 uses ACE2 as its entrance receptor and TMPRSS2 cell protease for S protein priming [20]. Cross human tissue overviews of ACE2 and TMPRSS2 positive cells discovered co-expression of these proteins in nasal goblet and ciliated epithelial cells and oligodendrocytes [21]. ACE2/TMPRSS2 co-expression in oligodendrocytes could be one of the ways for the virus to enter the CNS. Furthermore, lopinavir/ritonavir and azithromycin are used in the management of COVID-19. Their interaction with many common medications including antihypertensives, antiplatelets, statins, and anticoagulants has been reported in patients with prior strokes [22]. Lastly, these medications also pose a risk of neurocognitive impairment when used for longer periods [22].

In the light of the above-mentioned facts and results, COVID-19 could potentially have a long-term effect on the brain. This could possibly lead to impaired cognitive function, which in turn, would affect the quality of life. The study has its limitation as well. First, since the study was conducted in a single institute, the sample size was limited. Secondly, knowledge of past history of participants was limited. Third, participants were followed for a limited time duration. Therefore, future long-term studies are required to explore other neurological manifestations so treatment options could be adopted accordingly.

## Conclusions

Our study indicates that COVID-19 tends to produce a wide range of symptoms, ranging from headache to anosmia to increased risk of stroke, that complicates clinical management. Potential neurologic effects and drug interactions have been reported secondary to the medications used to treat COVID-19. At this point in time, the exact mechanism involved in the neurologic manifestations of COVID-19 is not clear. However, in light of the aforementioned facts, COVID-19 could potentially have a long-term effect on the brain and may lead to impaired cognitive function, compromising the quality of life. Therefore, while treating COVID-19, it is important that the clinicians must be aware of the potential neurologic complications. Lastly, proper follow-up, preferably for up to six months, should be ensured even after they have recovered. This would aid in timely recognition and management of the neurological disorder.
